# Unmasking recurrent melena as the initial presentation of metastatic prostate cancer: a case report

**DOI:** 10.3389/fonc.2025.1674548

**Published:** 2025-09-12

**Authors:** Xiao Wang, Lixia Wang, Kaiqi Sun, Ying Gao, Lai Wei, Zhijun Duan

**Affiliations:** ^1^ Department of Gastroenterology, The First Affiliated Hospital of Dalian Medical University, Dalian, China; ^2^ Department of Nuclear Medicine, The First Affiliated Hospital of Dalian Medical University, Dalian, China; ^3^ Department of Pathology, The First Affiliated Hospital of Dalian Medical University, Dalian, China

**Keywords:** melena, metastatic prostate cancer, initial presentation, atypical manifestation, diagnostic challenge, delayed diagnosis, case report

## Abstract

**Background:**

Prostate cancer, a common malignancy in the male population, is typically characterized by urinary symptoms, such as urinary obstruction and frequency. This report presents a rare case of metastatic prostate cancer that initially manifested with recurrent melena.

**Case presentation:**

A 70-year-old man with recurrent melena was admitted to the hospital. Laboratory workups revealed significant pancytopenia, as well as highly elevated serum prostate-specific antigen (PSA) concentrations. However, both upper gastrointestinal endoscopy and colonoscopy found no abnormal bleeding lesions. Further positron emission tomography/computed tomography (PET/CT) examination and pathological results from the bone marrow and prostate gland confirmed the diagnosis of prostate cancer and bone metastasis. After androgen deprivation therapy (goserelin) along with an androgen receptor antagonist (darolutamide), the patient’s serum total PSA level declined drastically to 0.01ng/ml, accompanied by an improvement in pancytopenia. During follow-up, he reported significant symptomatic improvement, such as the cessation of melena.

**Conclusion:**

When encountering male patients with recurrent melena as the initial presentation, clinicians should be vigilant about the possibility of metastatic prostate cancer. Moreover, a comprehensive evaluation based on multimodality evidence and multidisciplinary management can inform an individualized therapeutic plan for prostate cancer progression.

## 1 Introduction

Prostate cancer is one of the most prevalent cancers in the male population worldwide, and it is also a major public health concern (1, 2). In the initial stage, prostate cancer is usually confined to the interior of the prostate gland without metastasizing to surrounding tissues or lymph nodes, manifesting several obscure and non-specific symptoms, including urinary obstruction, frequency, hematuria, or fatigue. Prostate cancer typically metastasizes to several common sites, such as bones, lymph nodes, and lungs, along with concomitant site-specific metastatic symptoms. However, gastrointestinal manifestations, particularly presenting as recurrent melena, are highly rare in the initial symptoms of prostate cancer. Here, we outline a case of metastatic prostate cancer with the primary symptom of recurrent melena, which can be a teachable moment for the occurrence of delayed diagnosis of metastatic prostate cancer in the differential diagnostic procedures of melena.

## 2 Case presentation

A 70-year-old male was admitted on February 28, 2025, presenting with recurrent melena for 7 years. He reported tarry or tawny stool with an average frequency of about 2–3 times per week, along with dizziness, fatigue, and peripheral pain. During the disease, he had taken non-steroidal anti-inflammatory drugs (NSAIDs) due to peripheral pain. His previous medical history was a 7-year history of coronary heart disease, and he underwent percutaneous coronary intervention (PCI), after which he took aspirin regularly (100mg, once a day). The patient had no personal history of smoking or alcohol abuse, and no family history of hereditary diseases. Positive physical examinations showed pale palpebral conjunctivae, and other vital signs were normal.

Initial routine blood workups in the local hospital before hospitalization revealed: hemoglobin (Hb) 93 g/L (reference range, 130–175 g/L), platelet (PLT) 54×10^9^/L (reference range, 125-350 ×10^9^/L), and alkaline phosphatase (ALP) 1068 U/L (reference range, 45–125 U/L). Further laboratory tests, organized after hospitalization, are listed in **Table 1**.

**Table 1:** Laboratory assays of several indices.

Index	Reference range	Initial presentation (Day 1)	Re-presentation (Day 4-6)
White blood cell (×10^9^/L)	3.5-9.5	2.75	2.35
Neutrophil (×10^9^/L)	1.8-6.3	1.52	1.35
Lymphocyte (×10^9^/L)	1.1-3.2	0.86	0.73
Erythrocyte (×10^12^/L)	4.3-5.8	2.62	2.39
Hemoglobin (g/L)	130-175	78.00	71.00
Hematocrit (%)	40-50	23.90	22.00
Platelet (×10^9^/L)	125-350	76.00	81.00
Alkaline phosphatase (U/L)	45-125	1068.00	816.00
Gamma-glutamyl transferase (U/L)	10-60	65.00	62.00
Albumin (g/L)	40-55	37.30	36.20
Erythrocyte sedimentation rate (mm/h)	0-15	70.00	55.00
Prothrombin time (s)	9.0-13.0	12.60	13.40
Prothrombin activity (%)	70-130	78.00	68.00
Fibrinogen (g/L)	1.8-3.9	5.00	5.00
D-dimer (mg/L FEU)	<0.55		1.60
Fibrinogen degradation product (mg/L)	<5.00		5.84
Carcinoembryonic antigen (ng/ml)	0-5	2.18	
Alpha-fetoprotein (IU/ml)	0-5.8	2.36	
Carbohydrate antigen 125 (U/ml)	0-35	8.75	
Carbohydrate antigen 19-9 (U/ml)	0-30	10.41	
Serum iron (μmol/L)	10.6-36.7		31.50
Total iron binding capacity (μmol/L)	50-77		38.80
Unsaturated iron binding capacity (μmol/L)	19.9-45.6		7.30
Transferrin saturation (%)	33-35		81.00
Serum ferritin (ng/ml)	27-375		1410.34
Occult blood in stool	Negative	Weakly positive	
Total prostate-specific antigen (ng/ml)	0-4.1		1043.00
Free prostate-specific antigen (ng/ml)	≤1.0		>50.00
FPSA/TPSA ratio	>0.15		<0.15

**Figure 1:**
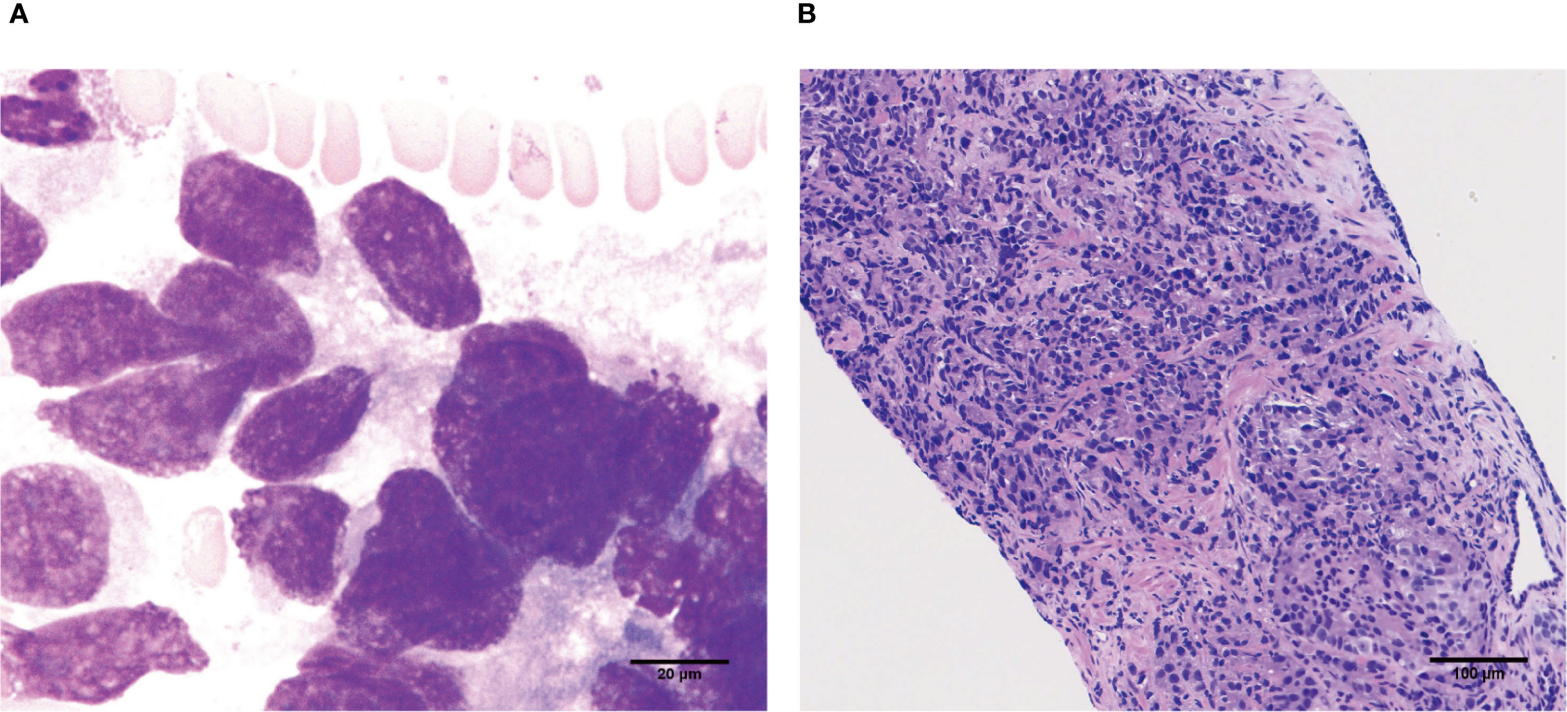
Pathological results from the bone marrow and prostate gland. **(A)** Bone marrow smear cytology showed unclassified cells, indicating possible metastatic bone marrow cancer (scale bar=20μm). **(B)** The pathological result of prostate aspiration biopsy demonstrated prostatic adenocarcinoma (Gleason score of 4 + 5 = 9, scale bar=100μm).

**Figure 2:**
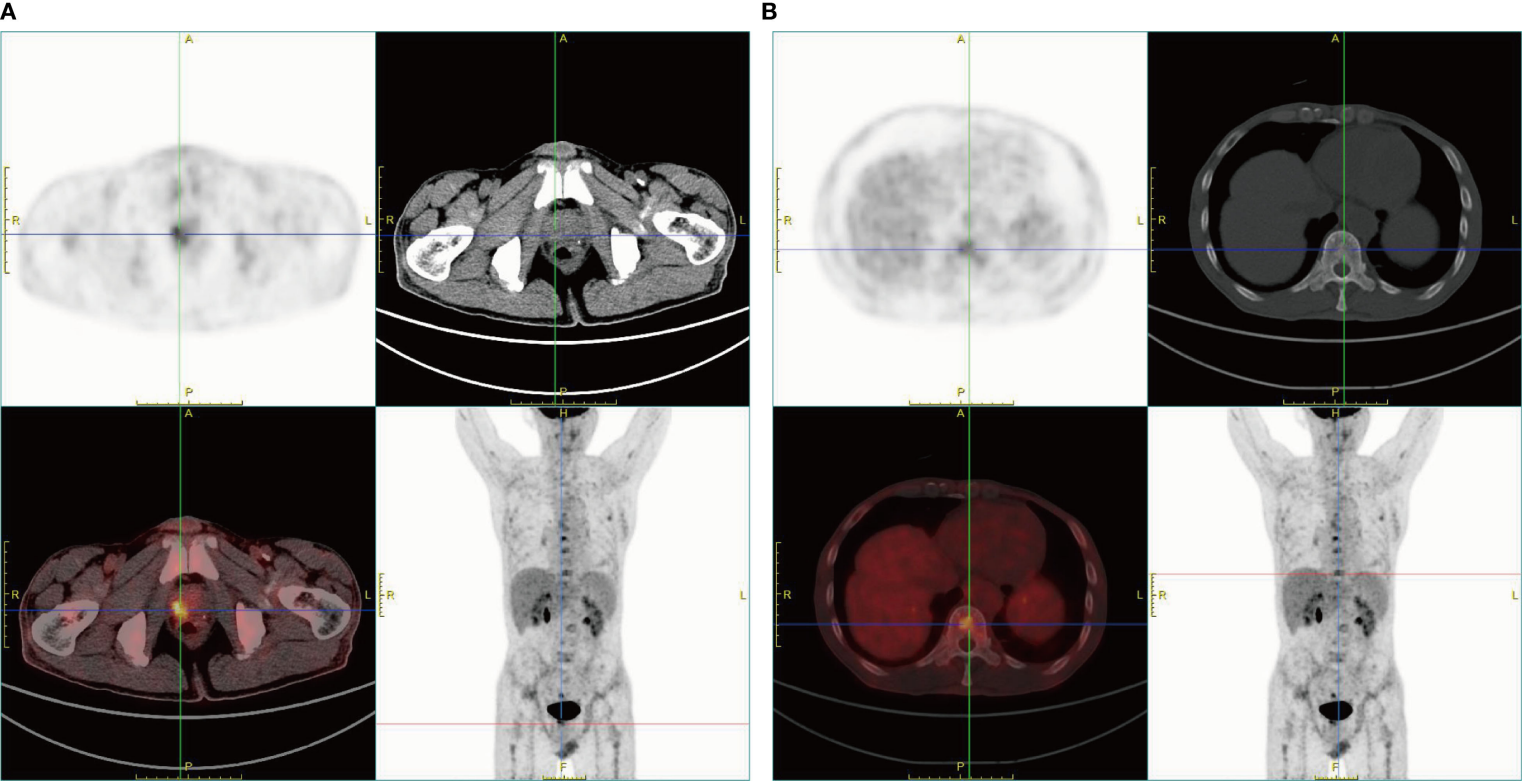
PET/CT images of the patient. **(A)** The enlarged prostate gland with punctate radiodense shadows within the gland was observed. A focus of increased FDG uptake was identified in the right lobe of the prostate gland, suggesting the potential of prostate cancer. **(B)** Diffusely increased bone density throughout the skeleton was observed. Foci of elevated FDG uptake in multiple bones were identified, suggesting the potential of bone metastases.

Considering medical history and laboratory workups, we sought to find potential clues of gastrointestinal bleeding. Abdominal contrast-enhanced computed tomography (CT) scan on admission exhibited a left liver hemangioma and prostatic hypertrophy with calcification. Upper gastrointestinal endoscopy and colonoscopy revealed chronic atrophic gastritis and a rectal polyp without apparent bleeding signs. Careful follow-up on the patient’s prior medical history revealed that the patient had taken non-steroidal anti-inflammatory drugs (NSAIDs) due to peripheral pain and PCI. However, the patient’s blood-related indicators were more severe in the re-presentation than in the initial presentation, with no evidence of active hemorrhage or an obvious lesion from endoscopy examinations. Therefore, the decision was made to complete further investigations. Notable biological indices revealed the following results: total prostate-specific antigen (TPSA) 1043.00 ng/ml (reference range, 0-4.1 ng/ml), free prostate-specific antigen (FPSA) >50.00 ng/ml (reference range, ≤1.0 ng/ml). Urinary tract ultrasound revealed a hypertrophied prostate gland (approximately 34×36×31mm) with calcification. Bone marrow smear cytology showed unclassified cells with possible metastatic bone marrow cancer (**Figure 1A**). Due to aberrantly increased serum indicators (such as ALP, TPSA, FPSA) and bone marrow pathology, a whole-body positron emission tomography/computed tomography (PET/CT) examination was subsequently performed. The scan revealed several regions with aberrantly increased fluorodeoxyglucose (FDG) metabolism in the right lobe of the enlarged prostate gland (SUVmax 6.2), along with diffusely increased bone density accompanied by elevated FDG metabolism in multiple bones (SUVmax 6.9). These findings suggested the potential of prostate cancer and bone metastasis, as presented in **Figure 2**. Then, the patient underwent an ultrasound-guided prostate aspiration biopsy under local anesthesia on March 12, 2025. The post-operative histopathologic results confirmed the diagnosis of prostatic adenocarcinoma, along with the Gleason score of 4 + 5 = 9 (**Figure 1B**).

Subsequently, the patient received androgen deprivation therapy (goserelin) along with a nonsteroidal androgen receptor antagonist (darolutamide). After 3 months of treatment, the serum TPSA level of the patient significantly decreased to 0.01 ng/ml, accompanied by an improvement in the pancytopenia. During the medical process, the patient reported an improved physical condition, with no melena and the alleviation of bone pain and fatigue. Moreover, no adverse effects were observed during treatment. The timeline of main clinical events in the diagnosis and treatment process of the patient is shown in **Supplementary Table 1**.

## 3 Discussion

Prostate cancer is the second-highest global incidence of malignant tumors in men worldwide (3, 4). The early symptoms of prostate cancer typically exhibit non-specific characteristics, such as dysuria and urinary frequency. Therefore, early and accurate diagnosis is often challenging. In this case, we reported a patient who was finally diagnosed with prostate cancer and bone metastasis, with recurrent melena as the initial symptom, which is relatively uncommon.

### 3.1 Reasons underlying recurrent melena in prostate cancer progression

The clinical manifestation of recurrent melena in prostate cancer is atypical and rare. A summary of previous cases associated with gastrointestinal bleeding and prostate cancer has been reviewed and organized in **Table 2**. Melena, a typical manifestation of upper gastrointestinal bleeding, can be caused by various etiologies, such as peptic ulcer disease and hemorrhagic gastritis. Further exploration of melena causes requires gastrointestinal endoscopy, which plays a crucial role in excluding primary gastrointestinal tumors and other overt lesions. In several previous cases, patients with gastrointestinal bleeding were always diagnosed with prostate-related benign or malignant lesions or presented with specific gastrointestinal hemorrhage causes (5–8). However, in the current case, the patient complaining of recurrent melena strikingly displayed no positive lesions for the explanatory evidence of gastrointestinal hemorrhage in both upper gastrointestinal endoscopy and colonoscopy. Careful follow-up on the patient’s prior medical history revealed that the patient took NSAIDs due to cancer-related peripheral pain and the postoperative need for PCI. According to this clue, we assumed that the pathogenesis of melena was partially attributed to the adjuvant administration of NSAIDs. NSAIDs function as a valuable pharmaceutical agent targeting cyclooxygenase to suppress prostaglandin synthesis, thereby reaching sufficient effects, including anti-inflammation and pain relief (5). However, NSAIDs can decrease the epithelial blood flow, reduce mucosal defense protective mechanisms, and impair platelet aggregative function, thereby further raising the risk of gastrointestinal adverse events, such as acute gastric mucosa damage and gastrointestinal bleeding. Therefore, the usage of NSAIDs might serve as a contributing factor in the pathophysiology of recurrent melena in this case.

**Table 2:** Summary of previous cases associated with gastrointestinal bleeding and prostate cancer.

Age	Case symptoms	Medical history	Main diagnosis	Gleason score	Treatment	Prognosis	Reference
61	Lower gastrointestinal bleeding, rectal pain, and psoriasiform dermatitis	BPH; hypertension	Metastatic prostate cancer (bone metastasis)	8	ADT, radiotherapy, and chemotherapy	Clinical improvement, including decreased rectal bleeding, resolution of skin lesions, and no adverse effects	(10)
76	Upper gastrointestinal bleeding and melena	Metastatic prostate cancer (bone metastasis)	·Metastatic prostate cancer (bone metastasis); ·Secondary duodenal carcinoma	Unknown	Unknown	Death occurred after 1 month due to septic shock	(11)
77	Epistaxis, hemoptysis, hematuria, hematemesis, and hemorrhagic shock	Unknown	·Metastatic prostate cancer (bone metastasis); ·Cancer-related DIC	7	·ADT, abiraterone acetate; ·Anticoagulation, platelet replacement, coagulation factors, and fibrinogen	Death occurred after 6 months	(12)
81	Altered mental status, aphasia, and left-sided hemiparesis, coffee ground emesis	Prostate cancer; hypertension	·Metastatic prostate cancer (bone metastasis); ·Gastric metastasis	Unknown	ADT, endoscopic hemostasis treatment	Death occurred after his family decided to withdraw care	(13)

BPH, Benign prostatic hyperplasia; ADT, Androgen deprivation therapy; DIC, Disseminated intravascular coagulation.

Increasing evidence has indicated that primary prostate cancer has a high potential for tropism to the bones. Bone metastasis can occur in approximately 10% of patients with early-stage prostate cancer and 80% of patients with advanced prostate cancer (6). In comparison with non-metastatic prostate cancer, metastatic prostate cancer contributes to dramatically greater incidence and mortality rates. Moreover, patients with metastatic prostate cancer always complain of bone pain, physical activity disorders, and are prone to pathological fractures, which may be due to the following reasons. First, cancer cells are inherently invasive. Once they infiltrate the bone marrow with narrow lumens, they can easily cause increased bone tension. Second, the aberrant occupation of malignant cells in the bone can further trigger dysregulated remodeling of the skeletal structure and destroy the bone cortex and bone marrow, contributing to bone pain. This can be confirmed by bone marrow morphology examination, which definitively acts as the gold standard diagnostic method (7). Given the unbearable bone pain, the use of NSAIDs for this patient might be reasonable. Additionally, metastatic cancer cells can have a profound impact on inhibiting normal bone marrow hematopoiesis, which can be a sentinel event and finally lead to significant thrombocytopenia, consistent with aberrant complete blood count examination in this case. It further collectively contributed to hemorrhagic tendency, which might also be a potentially important cause for the pathogenesis of recurrent melena, and a significant highlight of the difference compared with other similar cases.

### 3.2 Clinical diagnostic and management lessons for prostate cancer

Prostate cancer is often insidious in its early stages, which can gradually progress and deteriorate to develop life-threatening metastasis. Therefore, early detection and accurate diagnosis of prostate cancer with or without metastatic complications should be emphasized for determining the definitive staging of the disease, establishing prompt targeted treatment plans, and improving patients’ prognosis.

For early prostate cancer screening and detection, serum PSA is the most specific and sensitive tumor marker. The significant elevation in the serum PSA highly supports the existence of advanced prostate cancer, consistent with the current case. However, in this case, serum PSA was not always given priority for screening in the routine examination in the gastroenterology department, which might provide a reminder for gastroenterology doctors to enhance vigilance of the potential possibility of prostate cancer when encountering unexplained recurrent melena. Furthermore, laboratory results of serum ALP and ferritin can serve as potentially vital biomarkers for predicting dysregulated bone metabolism and monitoring prostate cancer progression (8).

Furthermore, several radiologic imaging examinations, including whole-body bone scanning, CT, magnetic resonance imaging (MRI), and PET/CT, usually play a fundamental role in assessing advanced prostate cancer and determining progressive stages. The different selection of imaging agents directly determines different sensitivity and specificity for the diagnostic procedures of prostate cancer. In this case, owing to suspicion of undefined tumors in the host and a previous history of PCI surgery, ^18^F-FDG PET/CT scanning, combining metabolic evaluation and CT imaging, was comprehensively considered and taken. It can sensitively detect possible tumorigenesis and bone marrow micro-metastases from solid cancers at the early stage, which may offer vital clues for carefully screening the potential possibility of unspecified cancers. Based on the clinical evaluation of this patient, ^68^Ga-prostate-specific membrane antigen (PSMA) PET/CT might be an alternative option for its accuracy and specificity in prostate lesions (9). Nevertheless, given that the initial ^18^F-FDG PET/CT findings were sufficient for the diagnosis of metastatic prostate cancer and economic factors were taken into account, the patient did not undergo additional PSMA PET/CT imaging.

Additionally, in the current clinical practice, the foremost challenge in treating patients whose first symptoms are non-urinary lies not in the diagnostic procedures themselves, but in the prompt recognition and comprehensive assessment of numerous potential etiologies and accompanying underlying diseases. For these patients, optimal management often requires a multidisciplinary approach, tailored to their individualized manifestations based on thorough clinical evaluation. Due to the complexity of metastatic prostate cancer, multidisciplinary management in this study can comprise a series of medical decision-making and clinical management options for patients, such as the formulation of personalized treatment and multi-stage follow-up plans, and reaching ultimate goals of improving prognosis and quality of life, which might provide further valuable insights and experience for reference in similar cases.

## 4 Conclusion

This case described a patient with metastatic prostate cancer who presented with recurrent melena as a rare initial manifestation. Timely identification of the exact etiology underlying recurrent melena was crucial, as it could be the first clue to gradually uncover the mask of the progressive diagnosis of advanced prostate cancer. This diagnosis was ultimately confirmed by various laboratory, radiological, and pathological evidence and required a multidisciplinary management approach.

When encountering male patients with recurrent melena as the initial presentation, clinicians should be vigilant about the possibility of metastatic prostate cancer. Additionally, other than metastatic prostate cancer, recurrent melena can occasionally herald other non-gastrointestinal malignancies, such as lung cancer. This established link between the upper gastrointestinal hemorrhage and an occult extra-gastrointestinal malignancy provides a critical teaching lesson for gastroenterologists in the relevant clinical practice: in the absence of common gastrointestinal causes, a broad differential diagnosis that includes metastatic malignancies is essential.

## Data Availability

The original contributions presented in the study are included in the article/**Supplementary Material**. Further inquiries can be directed to the corresponding author.

## References

[1] Bray F Laversanne M Sung H Ferlay J Siegel RL Soerjomataram I. Global cancer statistics 2022: GLOBOCAN estimates of incidence and mortality worldwide for 36 cancers in 185 countries. CA Cancer J Clin. (2024) 74:229–63. doi: 10.3322/caac.21834, PMID: 38572751

[2] Schafer EJ Laversanne M Sung H Soerjomataram I Briganti A Dahut W. Recent patterns and trends in global prostate cancer incidence and mortality: an update. Eur Urol. (2025) 87:302–13. doi: 10.1016/j.eururo.2024.11.013, PMID: 39668103 PMC11862828

[3] Bergengren O Pekala KR Matsoukas K Fainberg J Mungovan SF Bratt O. 2022 Update on prostate cancer epidemiology and risk factors-A systematic review. Eur Urol. (2023) 84:191–206. doi: 10.1016/j.eururo.2023.04.021, PMID: 37202314 PMC10851915

[4] James ND Tannock I N’Dow J Feng F Gillessen S Ali SA. The Lancet Commission on prostate cancer: planning for the surge in cases. Lancet. (2024) 403:1683–722. doi: 10.1016/S0140-6736(24)00651-2, PMID: 38583453 PMC7617369

[5] Obeng F Adamu AF Gavor SE Setsoafia BY Antwi EK Affram N. Case Report: Atypical prostate cancer presentation: rectal bleeding, pain, and psoriasiform dermatitis. Front Oncol. (2025) 15:1476988. doi: 10.3389/fonc.2025.1476988, PMID: 40206588 PMC11979128

[6] Bakry M Ahmadzai H Fadia M Peters G Kanjanapan Y Chitturi S. Cancer metastasis to the upper gastrointestinal tract-a case series. J Gastrointest Oncol. (2024) 15:2728–34. doi: 10.21037/jgo-24-532, PMID: 39816019 PMC11732330

[7] Giszas B Fritzenwanger M Grimm MO Stallmach A Reuken PA. Recurrent disseminated intravascular coagulation in metastatic castration-resistant prostate cancer: A case report. Diagnost (Basel). (2022) 12:2342. doi: 10.3390/diagnostics12102342, PMID: 36292031 PMC9600388

[8] Then EO Nutakki S Ofosu A Saleem S Gayam V Sunkara T. An unlikely culprit: gastric metastasis from primary prostatic adenocarcinoma. J Gastrointest Cancer. (2020) 51:1081–83. doi: 10.1007/s12029-020-00410-2, PMID: 32424673

[9] Stiller CO Hjemdahl P. Lessons from 20 years with COX-2 inhibitors: Importance of dose-response considerations and fair play in comparative trials. J Intern Med. (2022) 292:557–74. doi: 10.1111/joim.13505, PMID: 35585779

[10] Guruvayurappan GK Frankenbach-Désor T Laubach M Klein A von Bergwelt-Baildon M Cusan M. Clinical challenges in prostate cancer management: Metastatic bone-tropism and the role of circulating tumor cells. Cancer Lett. (2024) 606:217310. doi: 10.1016/j.canlet.2024.217310, PMID: 39486571

[11] de Visser KE Joyce JA. The evolving tumor microenvironment: From cancer initiation to metastatic outgrowth. Cancer Cell. (2023) 41:374–403. doi: 10.1016/j.ccell.2023.02.016, PMID: 36917948

[12] Cullis JO Fitzsimons EJ Griffiths WJ Tsochatzis E Thomas DW. British society for haematology. Investigation and management of a raised serum ferritin. Br J Haematol. (2018) 181:331–40. doi: 10.1111/bjh.15166, PMID: 29672840

[13] Gafita A Schroeder JA Ceci F Oldan JD Khandani AH Lecouvet FE. Treatment response evaluation in prostate cancer using PSMA PET/CT. J Nucl Med. (2025) 66:995–1004. doi: 10.2967/jnumed.124.268071, PMID: 40473460

