# Supplementary material for: Unmasking recurrent melena as the initial presentation of metastatic prostate cancer: a case report
Source: Front Oncol. 2025 Sep 12;15:1674548. doi: 10.3389/fonc.2025.1674548 (PMC12463589; doi:10.3389/fonc.2025.1674548)
Supplement: Supplementary file 1 [file Table1.pdf]

frontiers

Supplementary Material

1 Supplementary Tables

Supplementary Table 1. Timeline of clinical events in the diagnosis and treatment process of the patient.

Timeline	Event
February 28, 2025	• Hospital admission for recurrent melena.<br>• Abnormal hematological routine parameters and elevated ALP.<br>• Abdominal contrast-enhanced CT scan revealed no evidence of intra-abdominal malignancy.<br>• Gastroscopy and colonoscopy were scheduled.
March 3, 2025	Gastroscopy and colonoscopy identified no apparent bleeding lesions.
March 4, 2025	• Worsening pancytopenia and elevated ALP.<br>• Hematology consultation recommended further bone marrow puncture.
March 5, 2025	• Elevated serum ferritin.<br>• Bone marrow puncture was performed.
March 6, 2025	• ^{18}F-FDG PET/CT scan was scheduled.<br>• Elevated TPSA and FPSA levels.
March 7, 2025	• PET/CT findings indicated the potential of prostate cancer and bone metastasis.<br>• Bone marrow smear cytology suggested possible metastatic bone marrow cancer.
March 10, 2025	Urology and oncology consultations recommended further prostate aspiration biopsy for definitive diagnosis and treatment planning.
March 12, 2025	Ultrasound-guided prostate aspiration biopsy was performed.
March 13, 2025	The post-operative histopathologic results confirmed prostatic adenocarcinoma (Gleason score 4+5=9).
March 27, 2025	ADT (goserelin) with darolutamide was initiated.
May 5, 2025	TPSA 0.115 ng/ml.
June 23, 2025	TPSA 0.01 ng/ml, accompanied by an improvement in pancytopenia.
July, 2025	Follow-up: The patient reported an improved physical condition.

Abbreviations: ALP, Alkaline phosphatase; CT, Computed tomography; FDG, Fluorodeoxyglucose; PET/CT, Positron emission tomography/computed tomography; TPSA, Total prostate-specific antigen; FPSA, Free prostate-specific antigen; ADT, Androgen deprivation therapy.
